# High-dose benzodiazepine dependence: a qualitative study of patients’ perception on cessation and withdrawal

**DOI:** 10.1186/s12888-015-0493-y

**Published:** 2015-05-13

**Authors:** Michael Liebrenz, Marie-Therese Gehring, Anna Buadze, Carlo Caflisch

**Affiliations:** 1 Department of Psychiatry, New York State Psychiatric Institute, Columbia University Medical Center, 1051 Riverside Drive, New York, NY 10032 USA; 2 Psychiatric University Hospital, Lenggstrasse 31, 8032 Zurich, Switzerland; 3 Klinik Im Hasel AG, Hasel 837, 5728 Gontenschwil, Switzerland

**Keywords:** Benzodiazepines, Withdrawal, Patients’ perception, Qualitative study, Interview

## Abstract

**Background:**

Benzodiazepine withdrawal syndrome has been reported following attempts to withdraw even from low or therapeutic doses and has been compared to barbiturate and alcohol withdrawal. This experience is known to deter patients from future cessation attempts. Research on other psychotropic substances shows that the reasons and motivations for withdrawal attempts – as well as the experiences surrounding those attempts – at least partially predict future efforts at discontinuation as well as relapse. We therefore aimed to qualitatively explore what motivates patients to discontinue this medication as well as to examine their experiences surrounding previous and current withdrawal attempts and treatment interventions in order to positively influence future help-seeking behavior and compliance.

**Methods:**

To understand these patients better, we conducted a series of 41 unstructured, narrative, in-depth interviews among adult Swiss patients with a long-term dependent use of benzodiazepines in doses equivalent to more than 40 mg diazepam per day and/or otherwise problematic use (mixing benzodiazepines, escalating dosage, recreational use or illegal purchase). Mayring’s qualitative content analysis was used to evaluate findings.

**Results:**

These high-dose benzodiazepine-dependent patients decision to change consumption patterns were affected by health concerns, the feeling of being addicted and social factors. Discontinuation attempts were frequent and not very successful with fast relapse. Withdrawal was perceived to be a difficult, complicated, and highly unpredictable process. The first attempt at withdrawal occurred at home and typically felt better than at the clinic. Inpatient treatment was believed to be more effective with long term treatment (approaches) than short term.

Patients preferred gradual reduction of usage to abrupt cessation (and had experienced both). While no clear preferences for withdrawal were found for benzodiazepines with specific pharmacokinetic properties, participants frequently based their decision to participate in treatment on the availability of their preferred brand name and furthermore discarding equivalent dosage rationales.

**Conclusions:**

Our findings provide greater understanding of the factors that motivate high-dose benzodiazepine-dependent individuals to stop taking these medications, and how they experience withdrawal and treatment strategies. They underscore how patients’ perceptions of treatment approaches contribute to compliant or non-compliant behavior.

## Background

Benzodiazepines (BZD) are a highly effective psychoactive drug with anxiolytic, hypnotic, muscle-relaxant, and anticonvulsant properties. They are most commonly used to treat symptoms of anxiety and insomnia [1-3]. Adverse effects most notably include cognitive and psychomotor impairments, as well as dependence after continuous and/or long-term use [4-6]. Prevalence rates for long-term use among BZD users are estimated to be between 25-76% [7]. Furthermore, some 20 - 50% of BZD users are believed to experience some sort of withdrawal when trying to discontinue BZD after extended use, indicating signs of dependence [8,9]. While most long-term BZD users do not escalate dosage after reaching a saturation level, and remain within recommended dosage regimens, some patients develop high-dose dependence. Prevalence of this form of BZD dependence is difficult to estimate, but a cross-sectional study of the Swiss population found that 1.6% of patients with long-term use of BZDs (n = 25 354) received prescriptions exceeding recommended dosage by at least two times [10]. Matters are further complicated by a heterogeneous use of the term within the scientific community: Some authors differentiate between high-dose dependence that results from long-term prescription abuse following treatment of an underlying condition, and high-dose dependence that is a consequence of BZD use for recreational purposes (e.g., enhancing the effects of other drugs or reducing withdrawal symptoms, etc.) [11,12]. Regardless of group differences, however, high-dose BZD users are believed to suffer more frequently from comorbid mental disorders, might not sustainably benefit from current discontinuation and withdrawal strategies, and are thus exposed to an increased risk of impairment and injury [13-16]. Benzodiazepine withdrawal syndrome has been reported following attempts to withdraw even from low or therapeutic doses [12,17], and has been compared to barbiturate and alcohol withdrawal [18-20]. It has also repeatedly been associated with symptoms that can range from anxiety, panic attacks, sleep disorders, cognitive impairments, and muscle spasms, to perceptual hypersensitivity, depersonalization, hallucinations, excitability, symptoms of psychosis, and convulsions [20,21]. While the perceived severity of these symptoms has been linked to long-term and high-dose BZD use, fast-onset and short-acting BZDs, and anxious personality traits, it occurs very frequently, with an incidence of 30-100% (not taking into account the 50% of long-term BZD users who have been reported not to consent to withdrawal studies or to later pull out of them) [12,22,23]. Furthermore, the experience of withdrawal is known to deter patients from future cessation attempts. Accordingly, some researchers have called for investigation of long-term and/or high-dose users in relation to evaluating discontinuation of treatment [15,24,25].

Research on other psychotropic substances shows that the reasons and motivations for withdrawal attempts—as well as the experiences surrounding those attempts—at least partially predict future efforts at discontinuation, as well as relapse [26,27]. We therefore aimed to qualitatively explore these clinical questions within a sample of high-dose BZD-dependent patients to better understand patients’ perceptions of current treatment interventions.

## Methods

### Study design

To elucidate high-dose BZD users’ reasons and motivations for withdrawing from these medications, and their view of previous BZD withdrawal attempts, the authors conducted an exploratory qualitative study. For the study, only users who took BZDs for an extended period of time, for a dose equivalent to more than 40 mg diazepam per day, and/or those who had an otherwise problematic use of BZDs (such as mixing BZDs, escalating their dosage, using BZDs for recreational purposes, or obtaining BZDs by illegal means), were invited to participate. A series of 41 unstructured, in-depth interviews lasting for about 60–90 minutes were conducted by interviewers who had previous experience with one-on-one qualitative procedures and the treatment of substance-abusing individuals. All participants were assured complete confidentiality and provided their written informed consent, specifically to the digital recordings of the interviews. Zurich’s cantonal ethics committee approved this study.

### Participants

Patients who presented to the in- or out-patient units of the Psychiatric University Hospital Zurich between 2011 and 2012 with a diagnosis of high-dose BZD dependence (according to ICD-10), and who were at least 18 years of age and willing to give written informed consent, were invited to participate in the interviews. Exclusion criteria were defined as vastly insufficient language skills and acute intoxication. The full chart of each patient was made available by the clinic, including a complete biographical and psychiatric history and the patient’s diagnosis according to ICD-10. The members of our research group approached potential participants, who were identified by treating physicians. Interviews were then conducted outside the regular treatment setting to further ensure that participants freely expressed their own views and perceptions. They were assured that no information from the interviews would be given to treatment providers. Incentives were provided to both inpatient and outpatient subjects for their participation into the study.

### Sample

A mixed method of purposeful sampling and saturation sampling principles was used. To achieve greater variation of themes and motives, we recruited subjects from both general treatment settings and from units specializing in the treatment of substance- use disorders. The sample was also selected to provide diversity in relation to: (1) comorbidity and past clinical experience, (2) duration of high-dose-benzodiazpine use, (3) gender (m/f), (3) age and (4) occupational status. Recruitment of participants continued until saturation of data was reached. In total sixty particpants were contacted. Forty-one agreed to participate. Obstacles to study participation were seldom addressed by potential participants. Fourteen participants left the impression of being too ashamed to talk about the subject. Only in two instances did participants decline to participate because they perceived the amount of compensation (approximately the equivalent of USD 5) as inadequate. In three cases, potential participants agreed to be interviewed, but withdrew their consent during the interview – naming a lack of interest in the research topic.

### Interview

In accordance with recommended principles of conducting qualitative research, the interview began with narrative opening questions; however, a self-developed topic guide (vide infra) provided a flexible interview framework to explore beliefs that were not spontaneously covered in participants’ initial narrative. Special care was given to ask open-ended and neutrally worded questions to avoid eliciting socially desirable responses. In addition, appropriate nonjudgmental and non-leading probes were used to explore perceptions that were raised spontaneously by in patients’ initial narratives. We allowed the themes and motives identified in earlier interviews to be explored in the ones that followed, and combined the principles of maximum variation and complexity reduction to simultaneously widen the scope of results and examine previous assumptions.

### Data analyses

Data collection and analyses were conducted simultaneously until saturation had been reached. All interviews were conducted in Swiss German (an Alemanic dialect spoken in the “German-Speaking” parts of Switzeralnd) digitally recorded, using dictamus for iOS, and then transcribed verbatim into Standard-German, since Swiss German is not a “written language”. Potentially identifying information was removed and transcripts were assigned a code number. Mayring’s qualitative content analysis approach was used to evaluate findings [28]. This framework constitutes a controlled approach for empirical and methodological qualitative analysis. Instead of approaching the data with preconceived assumptions, the data were allowed to “speak for themselves.” Materials were coded using an inductive qualitative procedure. Categories obtained were discussed by the research team to validate ratings and achieve consensus on a biweekly basis. ML applied the final code, with confirmation of consistency through blind dual coding of transcripts with MG and CC. All researchers applying the codes had received training either as psychologist or as psychiatrists and had previous research experience with qualitative methods [29,30].

## Results

A total of 41 participants were interviewed. One participant passed away after having completed the interview (and having given informed consent) thus data were still included. Table 1 shows the clinical and sociodemographic features of the sample. Data presented are predominantly self reported (employment status, benzodiazepine use profile) supplemented and objectified with information from individual patient charts (current medication, ICD-10 diagnosis). The mean duration of benzodiazepine use was 8.2 years +/− SD 6.82 (median 5.0 years) with a mean diazepam equivalent dosage of 83 mg +/− SD 69 (median 70 mg). Participants with a high-dose benzodiazepine dependence according to our inclusion criteria, had a high probability of carrying at least one (36.6%) or more (39.0%) lifetime psychiatric diagnoses according to ICD-10. 21 (52%) participants had a past or current affective disorder (ICD-10 F3), followed in frequency by personality disorders (ICD-10 F6) in 34.1% and neurotic, stress-related and somatoform disorders (ICD-10 F4) 29.3%. Only a minority of subjects (9.8%) had experiences with no other psychotropic substances than benzodiazepines. The majority reported a past or current use of one (19.5%) or more (70.7%) substances, most frequently citing heroin (68%), alcohol (63%) and cocaine (53.6%). The heterogeneity of this sample is further underscored by its employment status: While 29.3% were employed at the time of the interview, 26.8% were not and 39% were recipients of a disability pension. Type of labor varied greatly between unskilled (exotic dancer), semiskilled (housepainter, bus driver) and skilled work (welder, nurse, cook, social worker).

**Table 1:** **Clinical and sociodemographic features of the sample**

		n	%
Number of participants		41	
Gender			
	Male	31	75.6
	Female	10	24.4
Duration of use			
	<5 years	14	34.1
	5-9 years	12	29.3
	>10 years	14	34.1
	Could not recall	1	2.4
Age of onset			
	<25	15	36.6
	25-39	18	43.9
	>40	7	17.1
	Could not recall	1	2.4
Employment status			
	Employed	12	29.3
	Not employed	11	26.8
	Retired	1	2.4
	Disability pension	16	39.0
	No data	1	2.4
Diazepam equivalent dosages			
	<50 mg	14	34.1
	50-99 mg	14	34.1
	>100 mg	13	31.7
Lifetime substance use except for benzodiazepines			
	None	4	9.8
	One	8	19.5
	More than one	29	70.7
Number of comorbid psychiatric diagnosis groups except substance use disorders (F2, F3, F4, F6, F9)			
	None	10	24.4
	One	15	36.6
	More than one	16	39.0
Comorbid psychiatric diagnosis groups except substanc use disorders			
	F2	1	2.4
	F3	21	51.2
	F4	12	29.3
	F6	14	34.1
	F9	1	2.4

### Participants’ reasons and motivations for withdrawing from benzodiazepines

Subjects discussed a variety of reasons why they wanted to withdraw from BZDs. But it was interesting that they generally addressed this topic only after they were specifically asked to; they often perceived the wish to stop as self-explanatory and without need of further elucidation. The process that led participants to decide to stop taking BZDs was found to involve a multifaceted interaction of different factors. We identified three major themes that were important in affecting the decisions of patients to change their consumption patterns: (1) concern about health, (2) the feeling of being addicted, and (3) external social factors.

#### Concern about health

The primary reported motivation to discontinue BZD use was concern about health. Participants were typically afraid of serious cognitive and physical impairments if they continued use.

*“And I am now 34 and have read in the Internet that they (BZDs) destroy internal organs, and that it can have devastating consequences when you are taking them for a long time.”*

*VP_04*

Commonly, subjects drew upon their own experience with the substance and said that they felt they had noticed deterioration of their memory after extended use.

*“I actually wanted to stop it for a long time, when I noticed that I developed problems with my short-term memory…”*

*VP_17*

*“You are getting a little dumb. You are doing things that you later regret. I, for example, was cheating on my boyfriend while I was using Dormicum® (midazolam). And then I got pregnant and had to have an abortion, just because I was using that.”*

*VP_37*

Participants often noticed these subjective memory impairments in their performance of daily tasks, which heavily influenced their decision to change consumption patterns.

*“I have stopped cooking at home because I forgot so many things, and then it was just burned. Then my children prohibited me to cook. They are anyway all day in the University, and eat there.”*

*VP_33*

In addition, some participants reported that although they did not share the view that their chronic and high-dose BZD use had negative effects on them, they had heard dramatic descriptions from colleagues or physicians about the consequences of such consumption patterns. In this context, a number of subjects stated that they had witnessed other people’s inpatient withdrawal attempts and had found them so disturbing that this in itself had contributed to their decision to stop using BZDs.

*“I think from a rational thinking perspective it affected my brain not very much…but I have talked to people who have been taking benzos for 10 or 20 years and they tell you about headaches and this and that. That is something that scared me.”*

*VP_34*

*“My doctor told me that you can develop a dementia from it and I don’t want to get demented.”*

*VP_29*

Only a few participants felt that using BZDs had negatively affected their mood and resulted in a loss of energy, citing this reason as a main factor in their wish to stop.

*“I decided for a withdrawal, because during the last couple of weeks and months they really pulled me down and I had not leisure time activity any more, because I just took a bunch of benzos in the morning. It took the form that I lost my momentum.”*

*VP_25*

Two individuals reported that they were brought by ambulance to the emergency room and had later been stabilized at the hospital. These individuals perceived that live-threating events had caused them to consent to transfer to an inpatient psychiatric unit, and had motived them to undertake withdrawal.

*“They (ambulance) had to come and get me at home on an emergency basis. It took four days in the hospital to bring me back to life and then I decided to come here (to the inpatient psychiatric unit).”*

*VP_02*

#### The feeling of being addicted

Another motivational factor for many participants was the feeling of being addicted and/or dependent on a psychotropic substance.

*“…and then I someday I noticed that I woke up in the morning and was already thinking about where to get Dormicum® (midazolam), and I understood during the last months, that I could not continue like that, that I had to decrease the use.”*

*VP_22*

Explanatory models with a strong moral connotation often accompanied this motive:

*“You have to prioritize in life what is important and what is not. I think it is very important in life not be dependent on anything, or on a pharmaceutical drug for that matter, but once you have started you have entered a vicious circle and it is difficult to get out of it.”*

*VP_04*

*“It is almost like being in love. Blindly. When you are in love you are blinded too. You are in love with this drug… I have not needed it before, why do I need it now. So, get rid of it!”*

*VP_35*

For less abstract reasons, participants perceived their high-dose dependence as limiting their freedom of movement, both in relation to traveling and to having the leeway to spontaneously make or change plans.

Participants who had a history of/or a current comorbid heroin use often drew comparisons to opioid dependence; some of them linked abstinence goals for BZD use with a desire to terminate opioid maintenance treatment, as well.

*“Because it is crap, when you want to go into a foreign country, you have to take a package (of tablets) with you, and in some countries, they can act really stupid. It is the same with Methadone.You have to have a letter from your physician with you, but then it is all right. In Europe it is generally not a problem.”*

*VP_07*

*“It is the same like being heroin addicted, I build my own prison, I can not spontaneously decide what I want to do, where I want to go… I always have to check that I have enough drugs on me…”*

*VP_13*

One participant reported to have attempted BZD withdrawal out of interest in the results and to evaluate his competence without this drug.

*“And I asked myself, if I wanted to withdraw BZDs and see for myself If I can handle (social and business pressures) without using them.”*

*VP_19*

#### Relevance of social and interpersonal factors

A major source of motivation to cease the BZD use resulted from external social factors. Participants who were interviewed during an inpatient withdrawal attempt most commonly mentioned this motive; and others said that their immediate family members, relatives, and significant others were often frustrated with them for using BZDs. Thus, external and interpersonal factors were cited as an important motivational influence for discontinuing BZD use.

*“And then my girlfriend told me that I was not myself yesterday, that I was a different kind of human being. And that I could not change. And that really hurt my feelings. And then I told myself: “I will not take benzos anymore!”*

*VP_14*

*“I was really stressing out my family, because I forgot what they have told me and then I asked the same questions again and then they told me: ‘You have asked twice already, or we have told you yesterday…’”*

*VP_28*

Within this context, participants said that they either wanted to please their partners because they themselves believed that their BZD use had a negative effect on their social interactions, or that they forced themselves to participate socially because they were told by people related to them that they would end the relationship if the subject did not seek treatment.

*“It became very problematic lately. My wife always told me: ‘You forget everything, I tell you something and you forget it.’ And early this year she told me: ‘It does not work like this. If it goes on like this, you will be so far down, that you will not find home one day. And she told me, ‘If you stay like this, then you are disturbed, then I will leave you and take the child. You have to go into the hospital.’ And I think she is right.”*

*VP_08*

Since participants were often unaware of their erratic behavior, they were sometimes video-filmed with mobile devices to make it possible for family members or colleagues to confront them. Participants often experienced these showings as very shameful.

*“And then of course I ran into problems with my girlfriend. I came home sedated, always, falling asleep at the table. She was ashamed of me when we went to have dinner in a restaurant. I had hooded eyes, my head on the plate. But I had the impression, that I was all right, that I was normal and I was asking what kind of problems other people had with me… She then took pictures of me with the cellular phone and showed them to me when I was still sober the next morning. I could not believe it. That could not be me. I was shocked. Really. Terrible.”*

*VP_30*

On a different note, it became apparent that many participants were also under enormous institutional pressure to suspend BZD consumption They commonly reported that living facilities intended to terminate housing agreements in case they continued use of BZDs.

*“…it was basically an obligation, a demand. I was told that either I go for inpatient withdrawal or I will get kicked out of the sheltered accommodation I live in… but I would have gone anyway, it maybe a good thing to do…”*

*VP_26*

Many participants with children thought that their continued BZD use might negatively affect their parenting abilities, and cited this as a factor in their motivation to quit. One mother revealed that her children had been placed into custody and that she was mandated to stop using BZDs if she wanted to be with them again, while another was afraid of this scenario.

*“My children were taken from me. We wanted to enter a mother-child facility, but they were not sure if I would still be taking benzos. They told me that I could not enter; initially I would have to get into a (psychiatric) hospital, so that they would be sure I was not taking any benzos. That is the most depressing thing: that my children were taken and (placed into custody).”*

*VP_38*

Other practical reasons to enter a withdrawal treatment included potential loss of a driver’s license, anxiety over losing disability compensation, financial considerations, and/or physicians’ threat to stop prescribing BZDs.

*“I am scared that the disability insurance will come under a lot of pressure and that society will not continue to show solidarity with ill people, maybe because social thinking is vanishing. I hope that I am wrong… I could imagine that mental disorders will be taken out of the catalogue… This is one reason I want to withdraw. Maybe this attempt will improve my health status, and I have a very bad one, to the point that I have a little chance on the job market… but I am scared that without benzos, anxiety, depression, and the obsessions will come back…”*

*VP_11*

*“…It is getting more expensive and I cannot find a doctor who is prescribing it to me, and in the ZOKL (outpatient treatment center) they don’t want to give it to me, either. I think from his (physician’s) side it is legitimate… he did not want to watch how I destroy myself…”*

*VP_34*

### Participants’ view on previous BZD withdrawal attempts – symptoms, helpful strategies, and outcome

Participants’ experiences stopping BZD use were much more heterogeneous, especially in relation to duration and quality of symptoms. Despite this, we were able to identify seven common motives and a number of repeated perceptions about quitting BZD use.

#### Withdrawal is frequent and not very successful

Most participants in this sample of high-dose dependent patients reported multiple previous attempts to quit BZD use. While treatment often resulted in a reduction of the amount of BZD used, and sometimes (self-proclaimed) months or even years of abstinence, the majority of this sample reported frequent relapse, typically after days or weeks.

*“I went a lot to my psychiatrist, to my general practitioner, looking for a way that it would work for me. But my doc tells me you can only do a withdrawal attempt in the hospital. But I have been to this hospital at least 20 times. It does not work. For example: I withdraw in here, and leave for home. Then it is all right for two or three months and then it starts again.”*

*VP_18*

*“I tried to stop five-six times by myself…*

*VP_12*

Subjects who abused BZD in high doses and were also dependent on other psychotropic substances usually distinguished between their attempts to withdraw from different kinds of drugs:

*“So, like withdrawal, just benzos withdrawal? I went six-seven times, and twice just because of benzos… ”*

*VP_24*

#### Withdrawal is difficult, complicated, and unpredictable

None of our high-dose dependent study subjects described cessation of BZD use as relatively easy or unaccompanied by only minor complications. To the contrary, the vast majority of participants regarded withdrawal as highly stressful, accompanied by a wide variety of symptoms whose onset and duration were difficult to predict and ranged from days to months. Most often, subjects compared withdrawal to an influenza infection: they experienced chills, weakness, headache, muscle pains, abdominal pain, nausea, vomiting, diarrhea, tachycardia, dizziness, and vision disorders. Others reported irritability, nervousness, restlessness, difficulty sleeping, symptoms of depression and anxiety, tickling sensations, dissociation, and a complete loss of appetite. Furthermore, subjects repeatedly described withdrawal-related seizures that had left them very worried. Participants who had also attempted withdrawal from opioids generally described stopping BZD use as a much more difficult task.

*“I have experienced very bad withdrawal, it shook me out of bed, I was twirling around, chill-shivering, ice cold…”*

*VP_09*

*“…because if you stop it…ah…then comes the withdrawal, then you cannot sleep anymore… and when it gets really crazy is when you experience vision difficulties… for example this sheet of paper… 1, 3 weeks ago I could not have read it.”*

*VP_10*

*“…Benzodiazepines can be really sinister. You take one tablet less and you seem to do just fine for a week or two and then comes crashing down a huge wave. In the end I was for one week on zero Seresta® (oxazepam), but just when I left (the hospital) the bad episodes hit…”*

*VP_16*

*“I had had extreme tickling in my legs. Especially when I was lying down. It is just like heroin withdrawal… I was screaming in pain. It is being said that (benzo withdrawal) is like an influenza, just 10 times worse, but an influenza is nothing in comparison…You can not sleep and you are twitching the entire time. I must have been screaming during the night, then they always brought me a Temesta® (Lorazepam) 2.5 mg, then it got better. It is really…you get scared of the blood in your legs. You want to ligate them, or hit them. It is so bad you can not describe it if you did not experience it yourself. And than of course the twitching…”*

*VP_38*

*“…Many people say that BZD withdrawal is much worse than methadone, for example. But I experienced that differently… I was just shaking and had one epileptic seizure after another…”*

*VP_05 (participant deceased)*

#### The first time takes place at home

In this group, most subjects reported attempting an initial withdrawal either alone at home or with some colleagues. For the most part, these attempts were planned. However, some subjects reported that they only became aware of their dependence because they experienced influenza-like symptoms and were told by other people that these symptoms might be associated with ending their use of BZDs. It is not surprising that these initial attempts were usually conducted without consulting a physician and without pharmaceutical support; users abruptly stopped taking the drug. Results varied. Some participants experienced symptoms so severe that they sought medical help within days, while others reported epileptic seizures but still considered abrupt withdrawal a very effective form of treatment.

*“Yes, and then I made this withdrawal. And I made it alone and I made it! I was laying in bed for three days nauseous, vomiting and with diarrhea, and then finished…”*

*VP_01*

*“When I did the withdrawal at home, there were days when you could not leave the house because you were shaking so strongly, so you just stayed in.”*

*VP_15*

*“And then I stopped from one day to the other, alone, at home, my husband was working back then. On the first day, I did not notice anything; on the second day, neither. But on the third day it started with shaking, nausea, in the beginning just light. I told myself that I can bear this, it would pass and I did not go to my psychiatrist, I told myself, I will handle this alone. This went on for two weeks and at the end of the second week I could not eat, nor drink, nor sleep nor do anything. I could not sit still and was running all the time through the apartment…and finally I could not bear it any longer and I went to see my psychiatrist.”*

*VP_12*

*“Then I thought, all right, today I will not take any benzodiazepines and wop…I noticed that I felt withdrawal, nervousness, shivers. And soon I figured I should do my first withdrawal attempt, Rohypnol® (flunitrazepam) withdrawal for that matter and had immediately my first epileptic seizure. And I immediately broke my nose… (A seizure) is something I did not have before. But since then I had a lot of seizures, mostly when I don’t take anything…”*

*VP_27*

*“…Ahh, and I did one benzo withdrawal with a friend of mine and my mother in Italy. My physician did not give me anything, but his doctor gave us tablets for the two of us. We went with 900 mg down there and had a party the first night. I had six packages with me, and I thought I will need that…”*

*VP_27*

#### At home feels better than in the clinic, but inpatient treatment is more effective

Participants expressed a clear preference for treatment approaches in an outpatient setting because they wanted to remain in their communities. However, they frequently pointed out that they did not manage to take their medication as prescribed when they were in the process of slowly decreasing. This often led to a decision to enter inpatient treatment—which was perceived as limiting personal freedom but was also considered faster and more effective because they found that BZD dosage was reduced more quickly in a hospital setting.

*“The entire withdrawal will probably take two-three weeks and until now it is good. I feel nothing. Actually I do not want to be here…and my psychiatrist wanted to send me here, but I did not want to leave my husband nor my dog alone, did not want to leave my home until it was almost too late…”*

*VP_12*

*“…I tried to reduce in an outpatient setting, but I did not make it, and that is when I said, ‘All right, I will enter the psychiatric hospital voluntarily for the withdrawal. But today it is a catastrophe, because it is too fast for my perception; we are reducing every other day…’”*

*VP_28*

*“Then you want to withdraw outside, but you don’t make it, because you start missing things (tablets). I think if it were close by, and I could receive the tablets just for each day, then I could get a better handle for it, as if I receive it for a week… it took a little time, but now I am here (inpatient unit).”*

*VP_34*

*“…My psychiatrist first wanted to do it in an outpatient setting, but that takes too long of a time because you have to reduce little by little dosages and that very slowly. But I have two little children at home, and I either function somehow or I am just out of the picture. It just does not work that I sit at home for three-quarters of a year. I knew what I was getting into. That is why I decided not for outpatient but for inpatient treatment. Better in the hospital, short and to the point…”*

*VP_41*

#### Gradual tapering is better than abrupt cessation, and few other things help

Participants tended to compare their different withdrawal experiences, and said it was easier to slowly reduce an administered dosage of BZDs. They expressed no clear preferences for BZDs with specific pharmacokinetic properties, but they did feel passionate about this subject and often extensively elucidated what worked for them. Because some participants had had favorable or unfavorable experiences with different brand names, they based their decision to participate in treatment on the availability of their preferred substance. These inclinations were not only highly subjective and often did not take into account equivalent dosage rationales—they also seemed sometimes to be uncorrectable by their physicians. Participants who mixed different BZDs considered the first days of treatment, and the search for an initial dose to taper from, as the most difficult part. Generally, subjects viewed neuroleptics as ineffective; and alternative, non-pharmaceutical approaches were rarely mentioned.

*“I had 12 mg Xanax® (alprazolam) a day, an incredible dosage. Over the period of one, one-and-a-half years, we weaned off it until zero…”*

*VP_29*

*“I am feeling very well. I did not make the same mistake like last time. That time I was reducing too swiftly, in the same time of two weeks I had already reduced by two tablets. That was too much…”*

*VP_16*

*“…when I see it is a good time to reduce the BZD dosage, then I will go down, but certainly not abruptly, fastly. I have done that in the past a lot, also with Methadone, and it then often proved to be counterproductive, that I took after the withdrawal even more than before. That is why I am telling myself: little, slow steps that are sustainable.”*

*VP_13*

*“… Valium® (diazepam) is the only thing that works against Dormicum® (midazolam). Seresta® (oxazepam) does not work. I have tried it. With this you are equally on withdrawal, even worse. In here they wanted to give me Seresta® (oxazepam) initially during admission. That is where I said: ‘No way! Otherwise I will just leave right now.’ Seresta® (oxazepam) just does not work in me. I then told them several times and then they said ‘O.K., then we will take Valium® (diazepam)…’”*

*VP_30*

*“They switched to Valium® (diazepam) so that we could do the withdrawal with Valium® (diazepam) (instead of Xanax® (alprazolam)). They initially made a calculation error, and I received far too little. One morning I was almost collapsing, but one of the nurses reacted very promptly. She gave me immediately the drug. I then sat down, took it, and two minutes late I was starting to feel better.”*

*VP_15*

*“…here in the inpatient unit, the first time, they were trying it with Seroquel® (quetiapine). That is a psychoactive drug, at first together with benzodiazepines, just less of them combined with that psychoactive drug. This (drug) did not show any effectiveness in me; it did not work how it was supposed to do.”*

*VP_13*

*“…I drink a lot of ‘Withdrawal Tea’ (a nursing staff mixture). I recommend that to everybody, I almost cannot taste it any longer but it helps, very good, for withdrawal, but today I think I will need more of the ‘chemistry’ (is referring to prescribed benzodiazepines)…”*

*VP_28*

#### Longer time spent in inpatient treatment is better than shorter

It was usual for study subjects to link a later relapse with the amount of time they had spent in inpatient treatment. Although many had experienced relapse days after leaving several months of inpatient treatment, they favored long-term treatment approaches over shorter interventions. In their search for these, some were even willing to sacrifice their employment. While several months of inpatient treatment were considered acceptable, long-term inpatient treatment in specialized facilities seemed not to be, since participants thought such interventions would alter their personalities.

*“And in the inpatient unit they withdrew me very slowly. I think I was there for three months. This went very well. And then I went for rehabilitation to another hospital, were I stayed for another two-and-a-half months… and then I was clean for almost five years.”*

*VP_12*

*“…Last time I was put under pressure by Dr. L. to get discharged (from dual diagnosis inpatient unit), because of my work position…But now I have terminated my employment and called Dr. K. (different inpatient unit) and asked him straightforward if I would be under time pressure and get kicked out after three weeks, or if I could do it in a way that I wanted, that I felt well…”*

*VP_25*

*“…I am scared of the admission to a long-term inpatient facility… How will it (therapy) change me? I am scared that I would lose my personality there, and become an entire ‘thing’ of psychiatry and psychology…”*

*VP_23*

#### Abstinence is the goal

Participants entered treatment with the clear expectation of complete BZD withdrawal and long-term abstinence. However, some viewed their dependence as chronic, after long-term use and frequent relapse, and were unsure if they could reach that goal.

*“…I have no doubt I will make it to zero… (though) I do take it for the last 15–20 years, always and always.”*

*VP_23*

*“…it is the question how realistic it is (to wean BZDs off completely) and if I can achieve it, but at the moment I think I should try it and see how it goes.”*

*VP_11*

## Discussion

Despite their clinical relevance, the reasons and motivations that high-dose BZD users decide to withdraw continue to be under-investigated. A recent study from Australia among current BZD users with unknown dosages identified “current lifestyle not okay” as the only category for patients’ reason to stop [31]. Through our study, we can add to this topic and report three major interrelating themes that lead people to withdraw from BZDs. First, participants described *health concerns*, most commonly in the form of cognitive and physical impairments. Second, subjects complained about the *feeling of being addicted* and said that BZD use presented them with a moral burden that limited their autonomy. Third (and motives from this theme were frequently mentioned), individuals intended to discontinue their BZD use because of external *social factors*. Participants were often exposed to pressure or even coercion from their relatives, or from institutional or governmental bodies, to change their consumption pattern.

These motives are not significantly different from the rationale provided by patients with other substance-use disorders [32-34]. However, to the best of our knowledge, our study is the first that explores these motives within a sample of high-dose BZD-dependent patients.

The themes that arose from the interviews conducted among this group also reflect general and deeply held views about discontinuation treatment and BZD withdrawal. On one hand, participants had a long history of abuse, had repeatedly attempted to stop taking this medication, and expressed dissatisfaction, disappointment, and frustration with the outcome (*withdrawal is frequent and not very successful*); on the other hand, many wanted to continue to withdraw completely (*abstinence is the goal*) and felt that it was well worth emotional and social sacrifices (*at home feels better than in the clinic*, *but inpatient treatment is more effective* and *longer inpatient treatment is better than shorter*).

The majority of high-dose BZD-dependent individuals indicated that BZD withdrawal symptoms were severe and presented a wide variety of clinical symptoms, found the duration of these symptoms difficult to anticipate, and commonly experienced prolonged post-withdrawal symptoms, as well (*withdrawal is difficult and unpredictable*, *with lots of complications*). More specifically, participants said that they had experienced chills, weakness, headache, muscle pains, abdominal pain, nausea, vomiting, diarrhea, tachycardia, dizziness, vision disorders, irritability, nervousness, restlessness, difficulties sleeping, symptoms of depression and anxiety, tickling sensations, dissociation, complete loss of appetite, and epileptic seizures, which are consistent with previous research on BZD withdrawal [35,36].

Patients with a comorbid opioid dependence similarly highlighted their perceived severity of symptoms and repeatedly described BZD withdrawal as more difficult than opioid withdrawal. This finding is in line with a previous semi-quantitative study [37]. The present study further demonstrates that many high-dose BZD-dependent patients—whether or not they had been prescribed BZDs by a physician—initially tried withdrawing without seeking medical advice, usually by abruptly stopping BZD usage in a home environment (*the first time happens at home*). Results varied, but a recurrent response in this study was the feeling that this had resulted in perceived epileptic seizures [38-40]. Alarmingly, some subjects viewed abrupt discontinuation as a very effective form of treatment even after experiencing such symptoms. In addition, even long-term high-dose users who had been prescribed BZDs evidenced a variety of misconceptions and lack of knowledge about the adverse effects of these drugs. For example, some participants said that they were surprised by their influenza-like withdrawal symptoms, did not associate them with abrupt discontinuation of BZDs, and had needed third parties to explain that they might be experiencing symptoms of dependence. These perceptions further illustrate the need to provide patients with comprehensive information on the benefits and risks of BZDs when initiating habit-forming treatment approaches, even if intended only for short-term use [41,42].

Participants favored gradual and long-term dosage tapering to abrupt withdrawal (*gradual tapering is better than abrupt stopping*, *and few other things help*). They thereby confirmed the benefits of a treatment approach that is in line with current recommendations and guidelines for therapeutic-dose users [12]. However, most participants in this sample who had a history of mixing BZDs were switched over at the time of admission to a single BZD with an elimination half-life of 4–20 hours (Lorazepam, Oxazepam), or, less frequently, to Diazepam (20–100 hours), which was then tapered off. Subjects especially perceived this initial dose finding upon admission as very confusing, and often exhibited a limited understanding of equivalent dosage calculations. As a consequence, they subjectively associated more severe withdrawal symptoms with different brand names rather than with insufficient dosage. In some cases, the treating physician’s choice of BZD for withdrawal contributed in a major way to the participant’s decision to engage or not to engage in treatment. We therefore recommend that physicians consider this finding, since it further underlines how subjective perceptions of treatment approaches contribute to compliant or non-compliant behavior, and thus to outcome in medical care [43,44].

### Limitations

Forty-one long-term, high-dose dependent patients in Switzerland were studied. At the time of interview, the majority were in inpatient treatment, so the authors do not claim that the study is representative for all high-dose dependent individuals. In addition, the sample recruited for this study was self-selected, so we probably missed individuals who felt uncomfortable discussing their BZD use and are therefore likely to have missed the views of those who felt especially sensitive about their BZD dependence. Although interviews were conducted outside the treatment setting and subjects were assured that no information (except suicidal ideation) would be made available to the treating physicians, some participants might have believed that interviewers were especially seeking their perceptions about the success of their current discontinuation therapy, and they might therefore have presented these perceptions. Despite these limitations, however, this study is, to our knowledge, the first exploratory study conducted among the subgroup of long-term high-dose dependent individuals with a wide variety of comorbid mental disorders.

## Conclusions

These findings provide deeper insights into the beliefs and views of high-dose BZD-dependent individuals, especially in relation to the factors that motivate high-dose BZD-dependent individuals to stop taking these medications, as well as how they experience withdrawal and current treatment strategies. Future research needs to address these important clinical questions within a larger and more diverse subject sample.
